# Improving Adherence to Prolactin Monitoring Guidelines in Acute Psychiatric Wards

**DOI:** 10.1192/bjo.2025.10348

**Published:** 2025-06-20

**Authors:** Hannah Campling, Kimia Ghaderi, Jayana Patel

**Affiliations:** North London Foundation Trust, London, United Kingdom

## Abstract

**Aims:** The purpose of this project was to improve clinician’s understanding and adherence to prolactin monitoring guidelines among doctors, reducing unnecessary testing and ensuring appropriate management of hyperprolactinaemia in patients on antipsychotic medications.

Prolactin monitoring in patients on antipsychotic medications is crucial for detecting and managing potential side effects. However, inconsistent adherence to monitoring guidelines can lead to missed diagnoses, unnecessary testing, and suboptimal patient management, inappropriate cessation of antipsychotics, or unnecessary addition of aripiprazole. This quality improvement project aimed to assess and improve clinicians’ knowledge and measure the adherence to prolactin monitoring guidelines across three acute psychiatric wards in a North London Hospital.

**Methods:** A retrospective audit examining prolactin monitoring practices of patient records from June–July 2024 was conducted across three inpatient wards in a North London Hospital. Key metrics included frequency of symptom inquiry, appropriateness of testing, and adherence to management guidelines. Following the audit, an educational intervention was implemented at North London Mental Health Trust Academic programme, consisting of a presentation and pre/post-teaching surveys to assess knowledge improvement. Flowcharts summarising the guidelines were subsequently displayed in doctors’ offices.

**Results:** The audit revealed low rates of symptom inquiry (7.4% in Sunflower, 6.25% in Tulip, 21.1% in Daisy) and high rates of unnecessary testing (44.4% in Sunflower, 81.25% in Tulip, 73.7% in Daisy) among patients on antipsychotics. Guideline adherence for managing raised prolactin levels was poor across all wards. The pre-teaching survey (n=37) demonstrated significant knowledge gaps, average 45% correct responses, particularly regarding age-specific monitoring and indications for testing. Post-intervention, a marked improvement in knowledge was observed across all domains in the post-teaching survey (n=16) with an average of 84% correct responses. For instance, correct responses regarding age-specific monitoring improved from 22% to 68.75% for women and from 11% to 81.25% for men.

**Conclusion:** This quality improvement project identified significant gaps in clinicians’ knowledge and adherence to prolactin monitoring guidelines. The educational intervention demonstrated substantial improvements in clinicians’ understanding of appropriate monitoring practices. Ongoing efforts, including the display of guideline flowcharts and plans for reassessment, aim to sustain these improvements. Future work will focus on measuring long-term adherence to guidelines and its impact on patient outcomes and resource utilisation.

